# Atlantoaxial Subluxation after Pyogenic Spondylitis around the Odontoid Process

**DOI:** 10.1155/2015/861403

**Published:** 2015-05-24

**Authors:** Atsushi Hasegawa, Mitsuru Yagi, Masakazu Takemitsu, Masafumi Machida, Takashi Asazuma, Shoichi Ichimura

**Affiliations:** ^1^Department of Orthopaedic Surgery, Kyorin University, Tokyo, Japan; ^2^Department of Orthopaedic Surgery, National Hospital Organization, Murayama Medical Center, Tokyo, Japan

## Abstract

*Study Design*. A case report and review of the literature. *Objective*. The aim of this study was to describe the conservative management of pyogenic spondylitis around the odontoid process. *Summary of Background Data*. Atlantoaxial subluxation after pyogenic spondylitis is rare. The therapeutic approach to infection of the upper cervical spine is controversial. *Methods*. Medical chart and radiological images of a 76-year-old male patient were retrospectively reviewed. Radiography revealed atlantoaxial subluxation, and an abscess was seen around the odontoid process on magnetic resonance images. Intravenous antibiotics and a halo vest were used to treat the patient. We then observed the patient's conservative treatment course. *Results*. C-reactive protein levels returned to normal 4 weeks after administration of the intravenous antibiotics. The patient's muscle weakness also completely recovered 8 weeks after administration of the intravenous antibiotics. Because the patient was able to walk without any support, surgical treatment was not necessary. *Conclusions*. Pyogenic spondylitis of the upper cervical spine is a rare manifestation. Surgical or conservative treatment must be selected carefully based on the patient's symptoms. If early diagnosis and treatment can be provided to the patients, conservative treatment can be achieved.

## 1. Introduction

Pyogenic spondylitis of the upper cervical spine is a rare manifestation. In 1896, Makins and Abbott first reported 2 cases of odontoid osteomyelitis [1]. Atlantoaxial subluxation (AAS) is a well-known complication of rheumatoid arthritis, trauma, and congenital disease. This paper presents a rare case of AAS after pyogenic spondylitis around the odontoid process. We considered conservative treatment or surgical treatment in such a case.

## 2. Case Presentation

**Figure 1:**
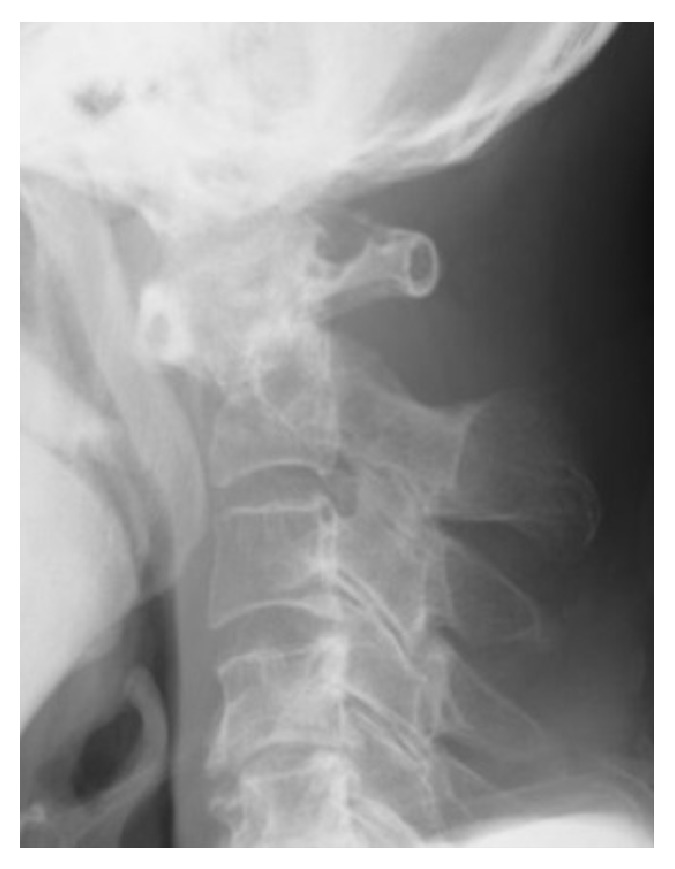
Lateral view of the cervical radiograph showing atlantoaxial subluxation in the flexion position.

The presented patient was a 76-year-old man whose chief complaint was persistent neck pain for two weeks. He had no significant past illness. He consulted a hospital, where his neurological status was found to be normal. After that, he suddenly developed quadriplegia and was transported to the nearby emergency hospital. His quadriplegia was dominant in the right half of the body and extremities. A radiograph of the cervical spine showed AAS (Figure 1). He was diagnosed with cervical spinal cord injury due to AAS and was then transferred to our hospital for the purpose of surgical intervention.

**Figure 2:**
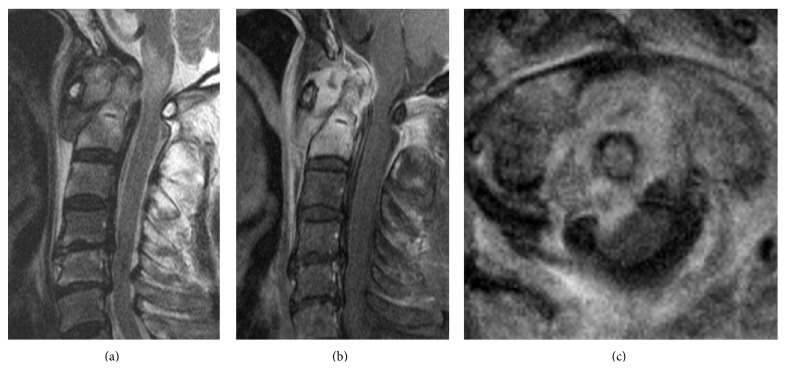
Magnetic resonance image showing abscess around the odontoid process. (a) Sagittal T2-weighted image. (b) Sagittal T1-weighted image with gadolinium. (c) Coronal T1-weighted image with gadolinium.

**Figure 3:**
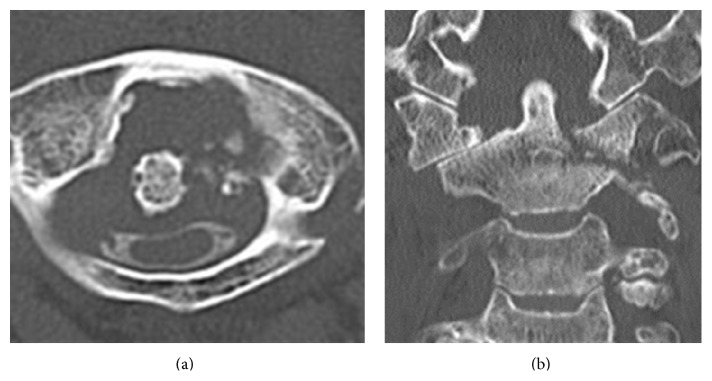
Computed tomography images after myelography showing destruction of the atlantoaxial joint on the left side and compression of cervical spinal cord. (a) Axial view. (b) Coronal view.

**Figure 4:**
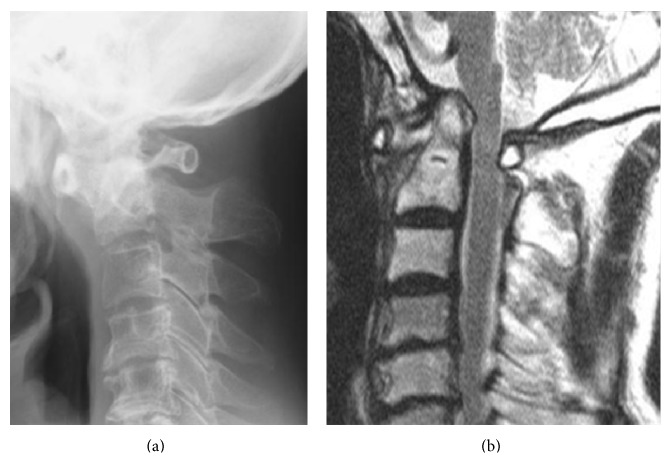
Images six months after the treatment of intravenous antibiotics and a halo immobilization showing vertical subluxation on the odontoideum and disappearance of the abscess. (a) Lateral view. (b) Sagittal T2-weighted image.

When he was admitted to our hospital, his body temperature was 37.2°C. His white blood cell (WBC) count and C-reactive protein (CRP) level were elevated to 14,200/mm^3^ and 20.2 mg/dL, respectively. Magnetic resonance imaging (MRI) with gadolinium enhancement showed an abscess around the odontoid process and an area of high signal intensity in the spinal cord at the C1/C2 level (Figure 2). Computed tomography myelography showed osteolytic destruction of the left atlantoaxial joint. The AAS developed because of destruction of the atlantoaxial joint and subsequent compression of the right side of the cervical spinal cord (Figure 3). His blood culture result was negative because he had been administered intravenous antibiotics (sulbactam/ampicillin) in previous hospital. The same intravenous antibiotics were administered, and a halo vest was applied in the same day. The WBC and CRP levels both returned to normal at 4 weeks after intravenous antibiotics administration, and his muscle weakness also recovered completely at 8 weeks after intravenous antibiotics administration. The WBC and CRP levels continued to be normal, and an MRI showed the abscess around the odontoid process had reduced in size. After 10 weeks, his halo vest was successfully removed, and he was discharged from our hospital without any assistance 12 weeks after the initial intravenous administration of antibiotics. At the follow-up examination after 1 year, an MRI showed disappearance of the abscess around the odontoid process (Figure 4(a)). A radiograph of the cervical spine still showed asymptomatic slight vertical subluxation, although this had not progressed (Figure 4(b)). We continued to carefully observe the patient and observed no recurrence of the infection.

## 3. Discussion

The AAS is a well-known complication developing secondary to congenital disorders, anomalies, trauma, and connective tissue disorders such as rheumatoid arthritis. The AAS is a serious condition that can lead to quadriplegia and sudden death due to cervical cord compression [2–5]. Pyogenic spondylitis of the upper cervical spine is seen in only 1.7% of all pyogenic spondylitis cases [6]. The AAS caused by pyogenic spondylitis is quite a rare manifestation. To the best of our knowledge, only 9 cases of AAS with neurological deficits secondary to pyogenic spondylitis have been reported (Table 1) [2, 4, 7–10].

Parke et al. reported that the upper cervical spine may be theoretically infected by congestion of effusion from the lymph and veins in the posterior pharyngeus. Spondylitis of the atlantoaxial joint can lead to loosening of the transverse ligament and subluxation of the joint [11]. Yamane et al. reported that approximately one-third of the cases reported before 1990 died. Earlier diagnosis with prompt treatment has resulted in recovery [8].

Several previous studies have reported that for patients with an infection that cannot be controlled with aggressive intravenous antibiotics treatment or for those with neurological deficits who cannot be stabilized by external fixation, curettage or decompression should be performed [3, 5, 6, 8, 12, 13]. In our case, the infection status and motor deficits both improved after the initial administration of antibiotics and halo vest fixation, and surgical intervention was not required. Due to the quite rare manifestation of AAS after pyogenic spondylitis, there is still an argument for surgical intervention for AAS secondary to the pyogenic infection. Morita et al. described that if instability of the atlantoaxial joint persists, surgical management should be considered [3]. On the other hand, Spies et al. reported a case that demonstrated destruction of the atlantoaxial and atlantooccipital joints on one side, and surgical intervention was not necessary [14].

Both in our case and the case reported by Spies et al., the patient was treated using external fixation with a halo vest; neck pain and instability of the joint gradually improved.

The management of pyogenic spondylitis around the odontoid process is still controversial. In general, pyogenic spondylitis with progressive paralysis will require surgical treatment. But if we observe the paralysis carefully in the hospital, where we can perform surgical treatment at any time, first treatment is local rest such as Glisson traction and intravenous antibiotics. If the treatment is effective, we change the traction to halo vest. But if the treatment is not effective, we must change the treatment to surgical treatment as soon as possible. In our case, we could successfully manage with conservative treatment. Earlier diagnosis and careful observation are important to avoid the need for surgical treatment for AAS after pyogenic spondylitis.

## Figures and Tables

**Table 1:** Case reports of atlantoaxial subluxation of pyogenic spondylitis around odontoid process with neurological deficits.

Number	Age/sex	Reference	Year	Past history	Bacteria	Operation	Outcome
1	72/M	Zigler et al. [7]	1987	Pneumonia	*S. aureus *	No	Stiff neck
2	41/M	Keogh and Crockard [2]	1992	Drug user	*S. aureus *	PSF	Recovered
3	61/M	Kim et al. [12]	2010	Unknown	TB	PSF	Recovered
4	68/M			Unknown	Unknown	PSF	Subjective weakness
5	64/M			Unknown	TB	Decompression	Subjective weakness
6	44/M	Bullock et al. [4]	2010	Retropharyngeal abscess	*S. aureus *	No	Stiff neck
7	83/F	Yamane et al. [8]	2010	UTI	MRSA	PSF	Walking with support
8	58/M	Yau and Li [9]	2010	No	*S. aureus *	Decompression + PSF	Mild numbness
9	45/M	Cheung et al. [10]	2013	Nasopharyngeal carcinoma	Unknown	No	Stiff neck

10	76/M	Our case		Nothing	Unknown	No	Stiff neck

Urinary Tract Infection (UTI); Posterior Spinal Fusion (PSF); *Staphylococcus aureus* (*S. aureus*); Methicillin-Resistant *Staphylococcus aureus* (MRSA); Tuberculosis (TB).
